# Ethical preparedness and developments in genomic healthcare

**DOI:** 10.1136/jme-2022-108528

**Published:** 2023-06-02

**Authors:** Bobbie Farsides, Anneke M Lucassen

**Affiliations:** 1 Clinical and Experimental Medicine, Brighton and Sussex Medical School, Brighton, UK; 2 Clinical Ethics, Law and Society (CELS), Centre for Personalised Medicine, Wellcome Centre for Human Genetics, University of Oxford, Oxford, UK

**Keywords:** Ethics, Genomics, Innovation, Preparedness

## Abstract

Considerations of the notion of preparedness have come to the fore in the recent pandemic, highlighting a need to be better prepared to deal with sudden, unexpected and unwanted events. However, the concept of preparedness is also important in relation to planned for and desired interventions resulting from healthcare innovations. We describe ethical preparedness as a necessary component for the successful delivery of novel healthcare innovations, and use recent advances in genomic healthcare as an example. We suggest that practitioners and organisations charged with delivering innovative and ambitious healthcare programmes can only succeed if they are able to exhibit the attribute of ethical preparedness.

## Introduction

 Rapid developments in genetic and genomic technologies over recent years promise massive transformations in healthcare with improved and earlier, diagnoses. Yet integrating these new technologies into healthcare also raise challenges for healthcare practitioners and policy-makers that stretch beyond the practical issues of cost, turnaround times and communication. In this paper, we explore what it would mean for healthcare practitioners and policy-makers to be ethically prepared to deliver these innovations and to meet the attendant challenges. While such stakeholders might easily default to a view of ethics as grounded in governance structures such as formal ethics committees, officially sanctioned rules and guidance, and conformity with the law, we argue that true ethical preparedness requires a much richer and more nuanced account of how ethics is incorporated into programmes of change and innovation. As such ethical preparation should run alongside the technical and infrastructure preparations, and ahead of the introduction or expansion of an innovative technology or change agenda. Moral work should both predate and continue long beyond the ethical approval stages provided by research ethics committees and/or regulatory bodies.

## Preparedness for what?

Many of us have become familiar with the concept of preparedness as a result of the recent pandemic and other forms of global crises, where emphasis is placed on being prepared in the sense of being ready to roll out and manage appropriate systems of service delivery in response to unexpected and unwelcome events^1^.

This coupling of preparedness with the ‘unwelcome’ is apparent in the literature of the COVID-19 pandemic.^2^ As recent experience has shown, even our most settled and uncontroversial services can be subject to swift and profound disruption—a volcano, a bank collapse or a newly mutated virus can challenge our most familiar institutions and undermine our sense of stability and security. Similarly, worldwide cultural movements such as Black Lives Matter or #MeToo can require us to re-evaluate and change practices to respond appropriately to welcome shifts in sociocultural and ethical perspectives. Appropriate handling of all these scenarios clearly requires a degree of ethical preparedness, yet we have often been caught unawares, suggesting the need to remain alert to the possibility that settled ways of doing things can be shown up as deficient or even harmful over time.

While recent focus on preparedness may have been prompted by our experience of unwanted upheaval and/or momentous social change, it has been our contention for many years that ethical preparedness is crucially important not just when responding to the unexpected, but also when developing and unrolling welcome and planned for change such as healthcare innovation.^3^

Given the assumption that healthcare innovation presents potentially valuable and welcome forms of change, the primary focus of preparation is often on practical and logistical issues. Yet, anticipating and planning for any ethical issues or challenges should also be a key part of preparation, and the potential for ethical challenge should not be underestimated. Some issues may be in plain view, or at least predictable from the outset, others may not become apparent until the innovation is implemented. One must ensure that healthy debate within relevant professional circles can not only emerge but thrive. Healthcare innovation will by definition place new responsibilities on healthcare professionals and expose patients and service users to new experiences, and these stretch beyond clinical and technical changes to new, or new aspects of ethical issues.

We suggest that clarifying and highlighting ethical issues, and revealing perceived difficulties in resolving them, is an important element of any pilot study or indeed a final feasibility or desirability assessment, and a necessary component of an effective implementation plan. Furthermore, as an innovative technology becomes more familiar, such as in the case of genomic medicine becoming increasingly mainstream, the responsibility to evaluate the ethical impact remains. An initial green light may change to amber or even red along the way. Indeed, an element of being ethically prepared to do something novel means not sidestepping the issue of whether we ought to be doing that thing—be that at all, or in the here and now.

## Preparedness for genomic medicine

While ethical preparedness can be explored and engaged in relation to a wide range of healthcare policies and interventions, here we focus on its pertinence to genomic medicine and will argue that the particular account we provide offers the possibility of successfully preparing for a future in which genomic investigations play a key part in prediction and diagnoses throughout the life course.

The study of genetics or heritability in medicine is not new, but up until the second half of the last century was limited to clinical diagnoses and recurrence risk estimation for children. Our ability to analyse the genetic code in cells improved exponentially from the turn of the century, with whole genome sequencing now affordable and quick enough for routine healthcare. Yet the enthusiasm about these technological developments can sometimes shroud the uncertainties that arise from genomic testing. For example, variants previously thought to be diagnostic when ascertained on the basis of disease, often prove only weakly linked when studied in a general population. We now know that genomic testing reveals that social and genetic relationships do not always align according to expectations, and we are also more aware of the fact that predictions about possible and untreatable diseases many years hence may not always be welcomed. The more genomic medicine reaches into other specialties the more practitioners grapple with ethical issues raised by the complex and open ended task of analysing our genetic ‘blueprint’, and they might find that off the shelf solutions are insufficient.

## Defining ethical preparedness

In its abstract form, we define ethical preparedness as a state from which one is able to identify and articulate ethical issues in a timely and ongoing manner, and where (ideally) one has the tools and the skills/experience available to address them. In the absence of the latter, one must know whom to consult and engage with in order to avail of appropriate expertise, a point we expand on below. While it would usually be preferable for individual practitioners not to delegate the responsibility to recognise and address ethical issues, we appreciate that there is a role for professional bodies, regulators and indeed patient groups to assist in alerting colleagues to their presence at an institutional and/or systemic level.

We consider that ethical preparedness is not a static state which one reaches and then freewheels within, but rather a dynamic response to challenges resulting from the introduction of healthcare innovations and/or shifts within established services. The edict to ‘be prepared’ is just as relevant when operating in familiar areas of activity where the ethically contested nature of the enterprise has been clearly established - examples might be abortion care, or assisted reproduction - as when dealing with new and unknown forms of practice. The concept of preparedness is necessarily relevant to areas of policy and practice where a degree of ethical challenge probably remains inevitable, and where the exact form that challenge takes is not necessarily predictable over time.

Preparedness is also more than a forward-looking attribute. Shifts in professional ethical standards and practices can result from a reassessment and critique of the activities of the past, some of which have resulted in the enacting of professional ethical standards we would not recognise as acceptable today. While we can justifiably critique those standards, and should reject any attempt to justify them in terms of ‘that was just how people behaved in those times’, we can also acknowledge and seek to better understand structural conditions that prevented individual ethical preparedness, and seek to rectify them.

Being ethically prepared, therefore, requires a willingness to engage with the past, to develop a nuanced understanding of the present context, and a commitment to think about and plan for possible futures. It requires a willingness to recognise and analyse failure as well as success. Consider for example, the work of the Retained Organs Commission and the Infected Blood Inquiry,^4 5^ both of which focused on the injustices and ethical shortfalls of the past, but both of which stand as invaluable exercises in preparing for a better and more patient-centred future. In the not too distant future politicians and policy-makers, and possibly individual practitioners could similarly be held to account for their response to one of the most seismic disruptions most of us will encounter in our life time, the COVID-19 pandemic, and we will need to be prepared to learn from this process in the same way.^6^

## Preparedness at different levels of policy and practice

As with many categories of action one can consider preparedness at a range of different levels, be that the preparedness of a national or supranational entity, a local institution or an individual. Clearly these become entwined and interrelated, with potential problems arising where preparedness at one level is dependent on another, or where an analysis of a situation shows a disconnect between, for example, the needs and perceptions of individuals and the institutions they operate within and under. So, for example, a health service practitioner is unlikely to be able to prepare fully for the ethical challenges of a new or amended practice without the support of their immediate employers, the wider healthcare system, and in cases where national policy is driving the endeavour, for example, the UK’s Department of Health and other relevant National Health Service (NHS) bodies.The challenge is to find a way of building preparedness that accurately maps the relationships between these different stakeholders and which focuses appropriately on the needs and capabilities of those closest to service delivery.

An important element of our account of preparedness is the requirement for working in close proximity to practitioners. Previous work both in relation to genetics,^7^,^10^ and more recently to genomics^11 12^ demonstrates that the day-to-day problems encountered by those charged with delivering a service can be just as ethically challenging as those encountered by those designing, embedding and managing the infrastructure supporting the service.^13^

While research ethics committees might scrutinise pilot projects before they start, and ethics advisory committees increasingly exist in the setup stage of high-profile projects, experience has shown that their influence is not always clear and nor do they necessarily have reach or influence once a service is up and running—particularly if it moves away from a specialist provider into the general health service.^12 14^ Given this, those responsible for establishing new modes of practice have a responsibility to inform their work with a clear understanding of how the processes, checks and balances that they put in place when designing or introducing and then running the service over time will be experienced, and practices enacted by those who will be working in the clinic and in the laboratories supporting these services.^15 16^ Clearly, there is a link here with the tenets of implementation science, a link we will come back to below.

## Ethical preparedness: a practical example

A long-standing example of commitment to ethical preparedness can be seen in the UK Genethics Forum.^17^ Established in 2001, the forum arose from a 1-day meeting called to explore ethical issues in genetics. This meeting and its aftermath highlighted healthcare professionals’ need and desire to discuss the intricacies of particular cases. A regular national ethics forum was established at which anyone working in clinical genetics—or branches of medicine engaging with genetics—could present and discuss cases with the aim of sharing experiences and potentially working towards models of good practice.^18^

The Genethics Forum has met triannually ever since and has thrived through being a multidisciplinary grouping which has always welcomed those with an academic or policy-based interest in genetics to come and work with, listen to and learn from those delivering genetic and more recently, genomic medicine. A key feature of the forum is that the ethical issues are described and analysed in the particular context in which they have arisen, meaning potentially abstract issues such as breach of medical confidentiality arise in many different forms and rarely invite a singular off the shelf solution. Indeed, while many health practitioners initially attend in search of legal or professional guidance relevant to their case, they leave better enabled to do the moral work required of them.

By emphasising the importance of what matters ethically to healthcare professionals and their patients, and by encouraging discussion and deliberation over particular cases and/or interventions arising in carefully described contexts, the Genethics Forum has set the tone for a particular approach to promoting and enhancing ethical preparedness. This approach emphasises the value of working in close proximity to clinical practice, the importance of understanding scientific, medical and clinical context, the salience of the facts relating to particular cases, and the need to revisit and reassess what has been seen as ethical over time. The latter being particularly true when major change is mooted or underway, but also when practices have become routinised and practitioners are at risk of being dangerously uncritical. Ethical clarity is sought by discussing the particularities of their experience, appreciating the relevance of context, through hearing suggestions from others who have encountered similar situations, as well as recognising that certain issues are never going to disappear or be definitively resolved. Through a discursive and reflective process attendees develop a form of ethical preparedness that has little to do with knowledge of law or guidance.

Ethical preparedness is thus about being willing and able to assess the ethical facts of the matter in particular cases to ensure that overarching principles—be they professional regulations or legal requirements—remain relevant and defensible. It also requires building the confidence to work in the absence of such shared principles, or in contexts where they no longer seem defensible or morally relevant. Indeed, this characterisation of preparedness favours situated and particular consideration of ethical issues over appeal to high level principles.

This, in turn, means that many actors and institutions can (and should) contribute to the achievement of preparedness, and one should not overlook the potential role of bodies whose day to day work is dealing with legislative and regulatory frameworks. As the introduction of mitochondrial donation and transfer demonstrates, statutory and regulatory bodies such as the Human Fertilisation and Embryology Authority (HFEA) can choose to facilitate and support the preparedness agenda by encouraging and facilitating public debate while also providing expert advice to parliamentarians and policy-makers.^19^ Similarly, the work of the Human Tissue Authority in the aftermath of the Retained Organs Commission and the Bristol Inquiry illustrates the way in which such bodies can be charged with helping to remedy previously long-standing ethical deficits, and the challenges involved in doing so.^20^

Having acknowledged their role and contribution, our preferred account of preparedness will always challenge individual practitioners to appreciate the constraints and limitations such bodies operate under, and the extent to which they may be unable to steer or resolve all clinically situated dilemmas. So, for example, while the HFEA’s work in relation to mitochondrial transfer can be applauded as an excellent example of encouraging and supporting public debate, thereby growing preparedness alongside evaluating and disseminating scientific knowledge, in other areas practitioners have felt that the Authority’s regulatory approach has left them prone to becoming entrenched in standard operating procedures that do not permit an ethical response to unusual circumstances not obviously covered by these procedures.^21^ In such situations, the practitioner might be left dealing with a sense that the ethically right course of action is known but unattainable through no fault of their own. This again is something we need to prepare for, particularly in areas of medicine where actions are heavily regulated and governed by statute which will be inevitably slow to respond to shifts in moral thinking in clinical settings. Preparation here might involve closer interaction and discussions between regulatory bodies and those adopting context sensitive approaches such as the Genethics Forum described above.

## Implementation science and ethical preparedness

Given the rapid introduction of innovative diagnostic and treatment options proposed by genomic medicine, a well thought out and nuanced implementation plan seems key. The field of implementation science aims to enhance the uptake of research into clinical or public health practice and examines factors in addition to the effectiveness of the innovation itself, identifying and addressing barriers and facilitators, and aiming ‘to improve the adoption, uptake and sustainability of evidence-based health interventions’. The gap between research findings and implementation is often measured in years, if not decades^22^ highlighting that establishing the effectiveness of a clinical innovation is not always sufficient to guarantee its uptake into routine use. Improvements in our abilities to delineate the sequence of a genetic code accurately, quickly and cheaply, has led to an expectation that this implementation gap can be reduced in genomics. Yet when Roberts *et al* reviewed the application of implementation research to genomics they found limited engagement with the opportunity to turn genomic developments into population health benefit. They identified a ‘need to apply implementation science principles to genomic medicine in order to deliver on the promise of precision medicine.’^22 23^ Recent research highlights a key implementation hurdle of general practitioners feeling poorly equipped to take on the ethical as well as clinical challenge of genomic medicine and a lack of confidence that measures are in place to remedy that deficit.^15^

## Ethical preparedness and the economic and political landscape

Our view is that being able to understand the nature and impact of organisational and societal landscapes within which key stakeholders are operating is key to developing ethical preparedness. In relation to genomics, it is important to understand the various ways in which our current landscape is infused with genomic activity, and the political and economic motivations behind this. The links between the genomic agenda and the UK’s industrial strategy are explicitly acknowledged, and the political enthusiasm for this area of medicine is at least in part due to its global marketability, and the opportunities it provides for partnerships that go far beyond traditional NHS models. It would be naïve to ignore this reality when considering why, when and how innovation is undertaken.

UK governmental support of the genomics industry^24^ has recognised the need for public support and engagement and work undertaken has demonstrated cautious public support.^25^ However, such support can be fragile. While resisting the invocation of slippery slopes, it is nonetheless important to consider what lines if any can be drawn to separate specifically health related genomic endeavours from other activities relying on the same technology and data. Immediately after announcing a newborn genomic sequencing programme (in which ‘up to 200 000’ babies would be offered their entire genomic sequence) the government released a report entitled Genomics Beyond Health: Preparing for a Genomic Future^26^ which laid out some of the applications of genomic testing out with the health service.

The report raised some consternation in informed circles, most importantly among those who had already donated their genomic data for medical research and clinical purposes who now wondered what other uses might be on the horizon. Genomics England responded to this concern emphasising the differences between their role and agenda, and the possible projects outlined in the report. They were careful to contrast speculative work from real time work such as their own stating:

….most of the topics and possibilities flagged in *Genomics Beyond Healthcare* are deliberately being highlighted *before* they have been validated scientifically, proven at the level of engineering or product, or brought into real world use.^27^

Add to this the unveiling of the innovative and ambitious Our Future Health initiative, which seeks to be ‘the UK’s largest ever health research programme, bringing people together to develop new ways to prevent, detect and treat diseases’^28^ and it is clear that we will need to be prepared to deal with genomics becoming part of our lives to a much greater and fuller extent in years to come. In recognising this fact it is also important to acknowledge that our genetic data is no longer the sole preserve of the clinic or the hospital based laboratory. With the proliferation of direct to consumer testing possibilities some of the principles guiding both policy and practice, and the assumptions that they are based on, are challenged and/or rendered obsolete. An example of how this can play out is demonstrated by the HFEA recognising a need to revisit its approach to donor anonymity given the fact that information is no longer under their sole control and can be obtained either intentionally or unintentionally through other routes.^29^

Alongside this back drop of genomic ambition and focus, it is also important to consider the ethical challenges posed or exacerbated by the current state of the NHS. It is an unavoidable truth in 2023 that any ambitious health related intervention is being planned at a time when NHS services and workforces are still struggling with the impact of the COVID pandemic,^30^ a crisis of funding, and industrial action prompted by a range of serious concerns. Furthermore, shifting the question being asked of genomics from diagnosis to screening of newborn babies raises new challenges such as training new actors (eg, midwives and health visitors) to be involved in the communication and support of parents to make informed decisions about a much broader screening of their newborn than conventionally offered. At a more fundamental level we need to develop new understandings of what constitutes a genomic result when there are so many different potential diagnoses and prediction of later onset conditions that might be suggested through the data created by this endeavour. The genomic sequence is immortal—the particular sequence of a baby’s genetic code will remain the same throughout their lives- but its significance will shift and change in unpredictable ways.

If we further acknowledge, that there is complex moral labour involved in the introduction of new programmes, it is incumbent on us to ensure that healthcare professionals are not only prepared to do such work (both in terms of capability and motivation) but also that they have the opportunity, capacity and support required to do so.^31^

Although the newborn screening study is described as a pilot and still in the research phase the Cochrane team highlight that ‘… a pilot study must answer a simple question: ‘Can the full-scale study be conducted in the way that has been planned or should some component(s) be altered?’.^32 33^ While we would argue that the question of whether or not all relevant parties are ethically prepared to do the work is a crucial component of such a pilot, this is not necessarily understood or accepted.

A full and proper account of the current ethical landscape would need to go further and deeper in understanding, for example, how genomics plays out against a background of health inequalities and discrimination, the impact of attitudes to disability and impairment, and the understanding (or lack of one) of sociocultural beliefs and practices in a diverse society.^34^

## The way forward

Given the ongoing complexities related to genomic medicine including newborn screening, we need to create and support opportunities to explore, or engage with, the wider ethical issues raised by work in this space.

One could ask whether in preparing for future advances in genomic medicine, intractable ethical concerns should or could ever lead to a decision not to proceed. Given the political momentum behind this proposal it seems unlikely, but at the very least it is important to suggest a detailed evaluation of the ethical burdens if any further innovation is undertaken, and gaps in preparedness need to be identified and revealed, and ultimately combined with rigorous scientific and clinical data to assess the success of well-planned and ethically approved pilot studies.

It is significant that Genomics England established both an Ethics Advisory Committee and a Participant Panel at the outset of the 100 000 genomes project, and these groups worked tirelessly to ensure that procedural and systemic issues were considered through an ethical and patient focused lens. This activity also informed processes around consent, confidentiality, sharing of data and communication of results. What the committee could not and did not do in its early days was provide a forum for individual clinicians or representatives of front-line services to seek advice, support or clarification. To some extent, either by design or default, this role fell to the aforementioned Genethics Forum.

As far back as 2000 the Wellcome Trust funded the project entitled ‘Cross Currents in Genetics and Ethics Around the Millenium’ to look at the ethical challenges posed by the ‘new genetics’ and specifically in the context of antenatal screening and testing.^35^ Interestingly, a key finding of this project was that practitioners were not ready to discuss and plan for possible genetic futures due to their continuing disquiet around aspects of routine screening and testing.^36^

At some level this was an early indication that preparedness requires an element of timeliness, such that the issues feel real and imminent as opposed to distant and theoretical. This introduces a difficult balancing act between coming in too late, or going in too early and thereby failing to bridge the theory practice gap. Considering the imminent pilot study, it feels as if there is an urgency relating to newborn screening that needs to be acknowledged and acted on.

In assessing our ethical preparedness for this or any other genomic adventure it is important to acknowledge that strong political support and an absence of polarising social concern should not be seen as indicative of a lack on moral concern about an enterprise. Indeed, a good place to look for detailed ethical scrutiny is with those who are supportive but cautious, those who seek greater recognition of the nuances, and those who can all too easily be dismissed as naysayers. All of these voices have been captured by empirical studies within our wider project^37 38^ involving NHS staff in clinical genetics clinics and the wider NHS primary and secondary care sectors and laboratories. Ethical preparedness involves inhabiting the space between enthusiasts and pessimists about genomics, because it is here that genomic imaginaries can be honestly presented and implemented successfully into practice.

## Conclusion

The increasing use of genomic medicine within our healthcare system and the current proposals regarding newborn screening, require us to build genuine ethical preparedness within both the professional groups charged with designing and implementing the genomic agenda, and with the future patients, parents and healthy citizens whose engagement will be crucial to its success. Whenever we introduce a new way of working we need to realistically assess how staff will respond to what is being asked for them, including the extent to which they will feel motivated to apply their depleted and overburdened energies to this particular endeavour. The question of whether the time is right has to extend beyond whether the technology is ready to the far more challenging question of whether we are genuinely ready to use it, and whether the healthcare environment is ready to accommodate it.

It should be recognised that building ethical preparedness is sometimes an unpredictable and potentially fraught process, particularly in the eyes of those who are most anxious for a project to proceed. In order to ensure ethical preparedness, one has first to identify and reveal ethical concerns and obstacles. Spaces and opportunities need to be created to explore and hopefully circumvent them, and in the rare and most extreme instances the discussions and strength of feeling in these spaces may call into question the very feasibility of the project being proposed. For a workforce and patient body to be properly prepared those advocating for and implementing change need to go beyond the committee rooms and policy hubs central to the endeavour, and when doing so they must ensure that engagement is not reduced to a form of conscription to the cause, or a trust building exercise. It is possible to be committed to an endeavour while at the same time being committed to being open about its challenges and limitations. An ethically prepared workforce will by definition be alert and concerned, but this should be seen as an asset rather than a danger. A disregarded and unprepared workforce may well prove to be the Achilles heel of an otherwise well thought out and implemented proposal. By giving healthcare professionals the space, permission and support to prepare themselves ethically for this work we do both them and their patients a great service.

## Data Availability

No data are available.
